# A *Malus* Crabapple Chalcone Synthase Gene, *McCHS*, Regulates Red Petal Color and Flavonoid Biosynthesis

**DOI:** 10.1371/journal.pone.0110570

**Published:** 2014-10-30

**Authors:** Deqiang Tai, Ji Tian, Jie Zhang, Tingting Song, Yuncong Yao

**Affiliations:** 1 Department of Plant Science and Technology, Beijing University of Agriculture, Beijing, China; 2 Key Laboratory of New Technology in Agricultural Application of Beijing, Beijing University of Agriculture, Beijing, China; 3 College of Horticulture, Shanxi Agricultural University, Taigu, Shanxi, China; National Key Laboratory of Crop Genetic Improvement, China

## Abstract

Chalcone synthase is a key and often rate-limiting enzyme in the biosynthesis of anthocyanin pigments that accumulate in plant organs such as flowers and fruits, but the relationship between *CHS* expression and the petal coloration level in different cultivars is still unclear. In this study, three typical crabapple cultivars were chosen based on different petal colors and coloration patterns. The two extreme color cultivars, ‘Royalty’ and ‘Flame’, have dark red and white petals respectively, while the intermediate cultivar ‘Radiant’ has pink petals. We detected the flavoniods accumulation and the expression levels of *McCHS* during petals expansion process in different cultivars. The results showed *McCHS* have their special expression patterns in each tested cultivars, and is responsible for the red coloration and color variation in crabapple petals, especially for color fade process in ‘Radiant’. Furthermore, tobacco plants constitutively expressing *McCHS* displayed a higher anthocyanins accumulation and a deeper red petal color compared with control untransformed lines. Moreover, the expression levels of several anthocyanin biosynthetic genes were higher in the transgenic *McCHS* overexpressing tobacco lines than in the control plants. A close relationship was observed between the expression of *McCHS* and the transcription factors *McMYB4* and *McMYB5* during petals development in different crabapple cultivars, suggesting that the expression of *McCHS* was regulated by these transcription factors. We conclude that the endogenous *McCHS* gene is a critical factor in the regulation of anthocyanin biosynthesis during petal coloration in *Malus* crabapple.

## Introduction

**Figure 1:**
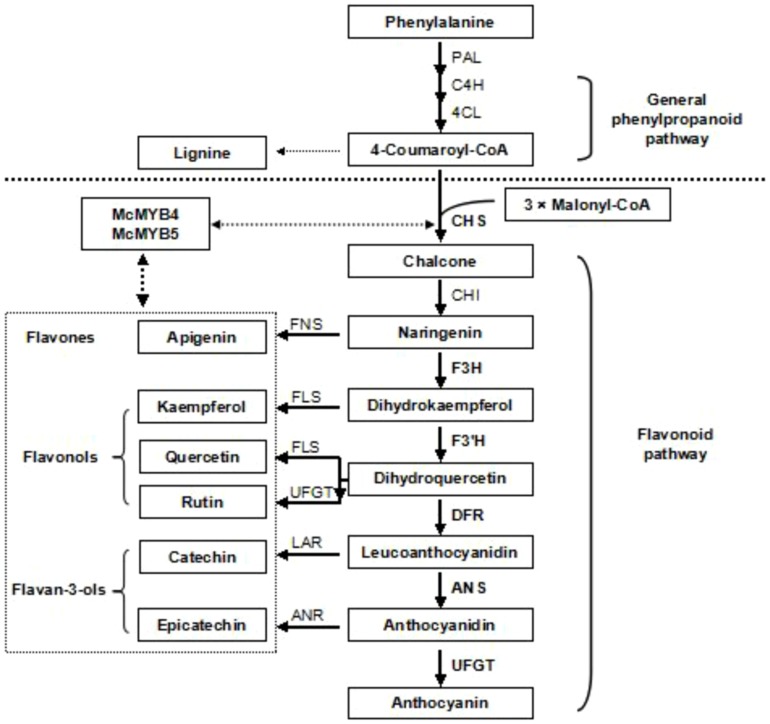
Scheme of the flavonoid biosynthetic pathway in plants. Genes encoding enzymes for each step are indicated as follows: *PAL, phenylalanine ammonia-lyase; C4H, cinnamate 4-hydroxylase; 4CL, 4-coumarate-CoA ligase; CHS, chalcone synthase; CHI, chalcone isomerase; F3H, flavanone 3-hydroxylase; F3′H, flavonoid 3′-hydroxylase; DFR, dihydro-flavonol 4-reductase; FNS, flavonol synthase; FLS, flavonol synthase; LAR, leucoanthocyanidin reductase; ANS, anthocyanidin synthase; UFGT, UDP-glucose: flavonoid-3-O-glycosyltransferase.*

The plant phenylpropanoid biosynthetic pathway leads to the formation of numerous compounds that are involved in diverse physiological and biochemical processes [1]. Some well-studied examples of these compounds include anthocyanins, flavonols and proanthocyanidins of the flavonoid family, which play a central role in the pigmentation of plant organs, seed germination, UV-B protection and defense against pathogens and biotic stresses [2]–[8]. Previous studies have focused on anthocyanin biosynthesis in *Arabidopsis thaliana* [9], *Petunia hybrida* [10], *Zea mays* [11] and *Malus domestica* [12]–[13], and anthocyanin biosynthetic genes have been characterized that are regulated by three classes of transcription factors (TFs): MYB, basic helix-loop-helix (bHLH) and WD40 proteins [14]–[16]. The reaction catalyzed by chalcone synthase (CHS) is thought to be the key regulatory step in the synthesis of flavonoids by catalyzing the condensation of one molecule of 4-coumaroyl-CoA with three molecules of malonyl-CoA to form naringenin chalcone, a major pigment of many flowers, leaves and fruits [17]–[19]. Indeed, chalcones provide the structural precursors for a broad range of flavonoids, flavonols, flavanones, anthocyanin glycosides and other derived compounds (Figure 1). Consequently, there has been much interest in *CHS* and its involvement in many aspects of plant physiology and biochemistry.

The first *CHS* gene was cloned from parsley (*Petroselinum crispum*) in 1983 [20] and, since then, numerous *CHS* genes have been isolated, mostly from monocots and dicots, including the legume soybean (*Glycine max*), alfalfa (*Medicago sativa*), pea (*Pisum sativum*), *A. thaliana*, barley (*Hordeum vulgare*), corn (*Zea mays*), grape (*Vitis vinifera*) and others [21]–[27]. CHS protein sequences are highly conserved among different plants [28], with amino acid homologies of approximately 80–90% [29], and molecular evolution analysis of *CHS* genes has shown them to be ubiquitous in plants, including early land plants and algae of the Charophyceae [30].

*CHS* has been well studied in the context of the synthesis and accumulation of anthocyanin pigments and several reports have described the effects of altering *CHS* expression in transgenic plants. For example, expression of an antisense *CHS* gene in petunia resulted in flowers that were pale colored, or even white, due to an inhibition of anthocyanin production, and plant fertility was also affected [17], [31]. Similarly, fruits from *CHS*-silenced strawberry (*Fragaria* × *ananassa*) lines were reported to have a lighter pink/orange coloration and a significant decrease in the levels of all flavonols, proanthocyanidins and anthocyanins [32]. As another example, fruits from apple (*Malus domestica*) *CHS*-RNAi knockout lines were shown to have no detectable anthocyanins accumulation and substantially reduced levels of dihydrochalcones and flavonoids [33].

*Malus* crabapple has one of the most economically important sets of ornamental apple germplasm resources. This represents a valuable source of research material, since crabapple exhibits excellent stress resistance and is useful for investigating the mechanism of plant pigmentation, due to the diversity of color in its leaves, flowers and fruits as a consequence of anthocyanins accumulation [34]. In this current study, we compared three crabapple cultivars: two extreme color cultivars, ‘Royalty’ and ‘Flame’ with dark red and white petals respectively, and an intermediate cultivar, ‘Radiant’, with pink petals [35]. To understand better the function of *McCHS* involved in the biosynthesis of anthocyanins and coloration level in the petals of these cultivars, we compared the expression of *McCHS* and other flavonoid biosynthesis pathway structural genes during petal expansion in these three typical cultivars by quantitative real-time PCR (qRT-PCR). Meanwhile, HPLC analysis provided an insight into the accumulation of anthocyanins and other flavonoids compounds in these different crabapple cultivars. The results suggest that the expression of *McCHS* in petals is well controlled, in a tissue- and developmentally specific fashion. We also overexpressed the *McCHS* gene in tobacco to evaluate its activity and the consequences of its expression. To sum up, *McCHS* expression is associated with petal coloration and the expression level of *McCHS* determined the coloration level of petals in different crabapple cultivars. Meanwhile, the expression level of this gene may be regulated by multiple MYB transcription factors.

## Results

### Petal phenotypes of three *Malus* crabapple cultivars, ‘Royalty’, ‘Radiant’ and ‘Flame’

**Figure 2:**
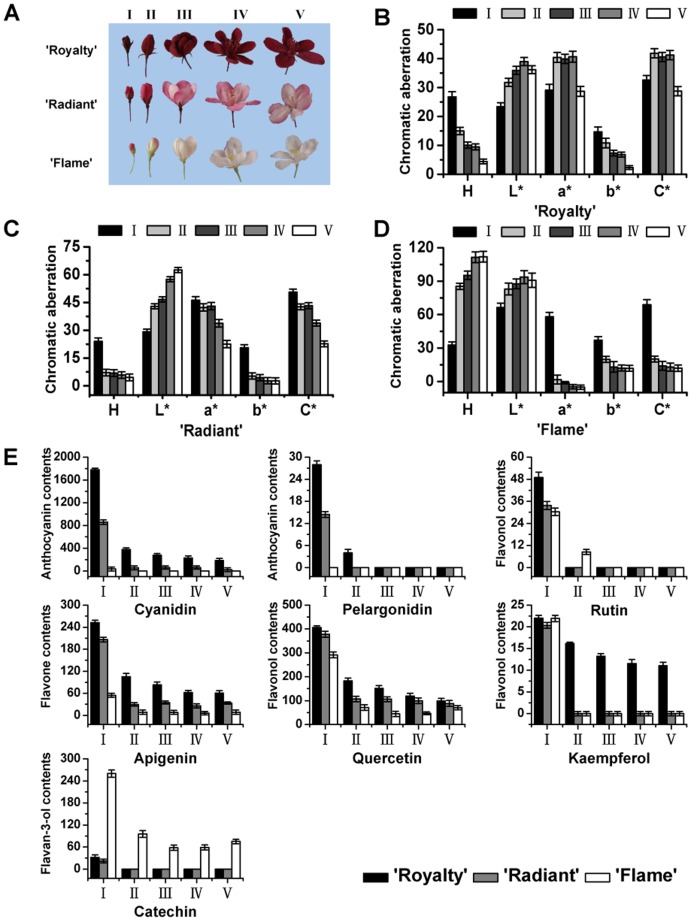
Flower developmental series of three *Malus* crabapple cultivars. (A) Typical flower phenotypes of *Malus* crabapple ‘Royalty’, ‘Radiant’ and ‘Flame’ cultivars through development. Five stages of each cultivar are shown. (B) Color changes in ‘Royalty’ petals. (C) Color changes in ‘Radiant’ petals. (D) Color changes in ‘Flame’ petals. (E) Content of flavonoids and anthocyanin in ‘Royalty’, ‘Radiant’ and ‘Flame’ petals. A spectrophotometric colorimeter was used to measure dynamic changes in petals, and HPLC was used to analyze the flavonoids and total anthocyanin contents. Five stages were tested in this study: (I) 6 days before full bloom; (II) 3 days before full bloom; (III) 1 day before full bloom; (IV) full bloom; and (V) 3 days after full bloom. Error bars indicate the standard error of the mean ± SE of three replicate measurements.

The petal phenotypes of three *Malus* crabapple cultivars, ‘Royalty’, ‘Radiant’ and ‘Flame’, are shown in Figure 2A. ‘Royalty’ petals at stage I were dark red and during flowering the color became more vivid and bright, ultimately reaching maximum color strength at stage IV of full bloom. At the later stage V, after full bloom, the pigmentation faded and dulled (Figure 2B). The petals of ‘Flame’ at stage I showed an obvious light red color, while from stage II to stage V, the color gradually became fainter and eventually the petals turned white (Figure 2D). Interestingly, the petals of ‘Radiant’ were red at stage I, and the color was gradually becoming pink during petal expansion (Figure 2C). Overall, ‘Royalty’ has the most red and vivid flowers, while the ‘Flame’ petals are almost white during petal development, and ‘Radiant’ petals have a significantly color fade process during petal expansion. Next, it is determined how the anthocyanins color the three typical cultivars.

### Quantification and identification of flavonoid composition during petal development in three *Malus* crabapple cultivars

To gain insight into the *Malus* crabapple petal flavonoid composition and its variation among cultivars, the levels of two anthocyanins (cyanidin and pelargonidin) and five other flavonoids were measured in the petals of the three cultivars (Figure 2E). Consistent with the petal color observations in the red cultivar ‘Royalty’, the abundance of anthocyanins was significantly higher than the other two cultivars, and a gradually decrease in the anthocyanins occurred in petal development, while anthocyanins were only detected in white cultiver ‘Flame’ petals at the first stage. Quercetin and apigenin showed the same spatial and temporal accumulation patterns, and the abundance of these compounds also decreased during petal development in all three varieties. Rutin had a similar profile to quercetin and apigenin and decreased substantially to undetectable levels except for in ‘Flame’ at stage II. In contrast, the catechin content of ‘Flame’ petal was much higher than petals of ‘Royalty’ and ‘Radiant’, and catechin was only found at the first stage of development in ‘Royalty’ and ‘Radiant’ petals. As the precursor for proanthocyanidin, the accumulation of catechin suggests that proanthocyanidin is the main flavonoids compound in colorless tissues and compared with other color-petal cultivars, the proanthocyanidin biosynthetic pathway is primary flavonoids biosynthetic branch pathway in ‘Flame’. In addition, the levels of kaempferol in ‘Royalty’ petals were almost same with those in the other two varieties in the first stage and decreased with the development of petals, the kaempferol contents in ‘Radiant’ and ‘Flame’ were barely detectable from the second stage to the fifth stage (Figure 2E).

### Characterization of the *Malus* crabapple *McCHS* gene

**Figure 3:**
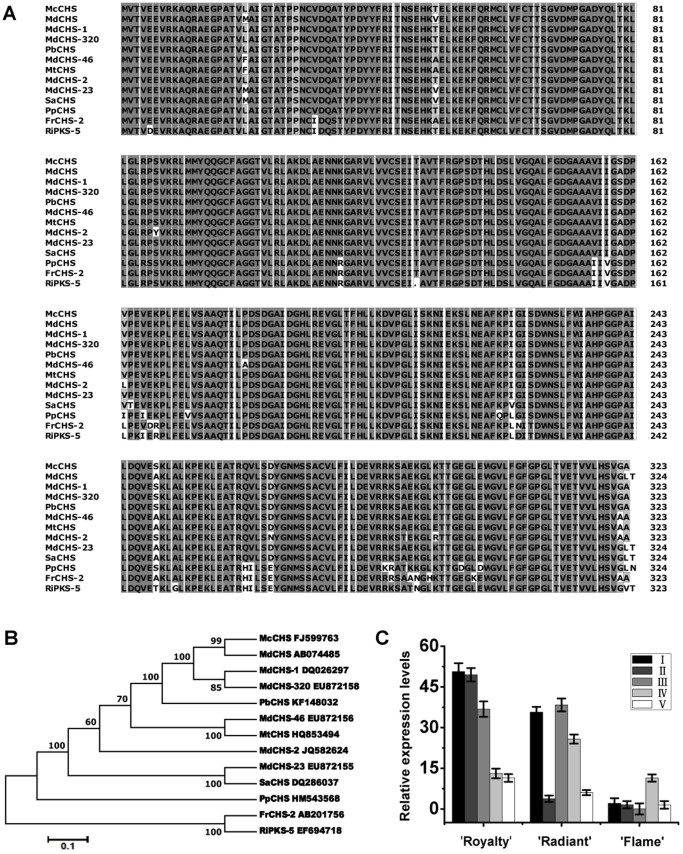
Sequence characteristics of McCHS and relationships with other CHS proteins. (A) Protein sequence alignment of McCHS and other known anthocyanin biosynthetic proteins from other plant species. Identical residues are shown in black, conserved residues in dark grey and similar residues in light grey. (B) Phylogenetic relationships between McCHS and CHS sequences from other species involved in anthocyanin biosynthesis. Phylogenetic and molecular evolutionary analysis was conducted using MEGA version 5.10 (using the minimum evolution phylogeny test and 1000 bootstrap replicates). (C) Relative expression profile of *McCHS* at different developmental stages of the petals of three *Malus* crabapple cultivars: ‘Royalty’, ‘Radiant’ and ‘Flame’. Five stages were tested in this study: (I) 6 days before full bloom; (II) 3 days before full bloom; (III) 1 day before full bloom; (IV) full bloom; and (V) 3 days after full bloom. Error bars represent the standard error of the mean ± SE of three replicate reactions. All real-time PCR reactions were normalized using the Ct value corresponding to a *Malus* crabapple 18S ribosomal RNA gene (DQ341382). The GenBank accession numbers of the proteins are as follows: McCHS (*Malus domestica*; FJ599763), MdCHS (*Malus domestica*; AB074485), MdCHS-1 (*Malus domestica*; DQ026297), MdCHS-320 (*Malus domestica*; EU872158), PbCHS (*Pyrus bretschneideri*; KF148032), MdCHS-46 (*Malus domestica*; EU872156), MtCHS (*Malus toringoides*; HQ853494), MdCHS-2 (*Malus toringoides*; JQ582624), MdCHS-23 (*Malus toringoides*; EU872155), SaCHS (*Sorbus aucuparia*; DQ286037), PpCHS (*Prunus persica*; HM543568), FrCHS-2 (*Fragaria ananassa*; AB201756), RiPKS-5 (*Rubus idaeus*; EF694718).

Based on homology with *Malus × domestica* sequences and related sequences in the Genome Database for Rosaceae (http://www.rosaceae.org), a gene sequence for the full length *CHS* (*McCHS*) was amplified from total RNA of *Malus* crabapple ‘Royalty’ leaves by RT-PCR and RACE. Specifically, the *McCHS* cDNA (accession no. FJ599763) is 1,529 bp in length and is predicted to encode a protein of 389 amino acids with a high level of homology to with *CHS* genes from a range of species (Figure 3A), including *Malus domestica* (MdCHS2; 99% identity), *Malus domestica* (MdCHS1; 99% identity), *Malus domestica* (MdCHS320; 99% identity), *Pyrus bretschneideri* (PbCHS; 98% identity), *Malus domestica* (MdCHS46; 94% identity) and so on. It appears that chalcone synthase protein sequences have undergone little sequence diversification in the species examined and that they are highly similar in length in different plant species. Other than the putative *CHS* gene sequences, there are also related sequences that are more divergent from characterized *CHS* genes and although these genes show high sequence similarity to a *Fragaria ananassa CHS* (FrCHS; AB201756), e.g. *Rubus idaeus* (RiPKS-5; EF694718; 97% identity), their functions have yet to be determined. Collectively they are described as polyketide synthase *(PKS)* genes, a more general description for the *CHS* and *CHS-Lik*e gene family (Figure 3B).

We performed qRT-PCR to determine the expression of *McCHS* during petal development of the three crabapple cultivars (Figure 3C). Compared with that in colorless cultivars, the relative expression level of *McCHS* in ‘Royalty’ was much higher, whereas *McCHS* transcripts were barely detectable in ‘Flame’ petals, except at stage IV. In ‘Royalty’ and ‘Radiant’ the transcript levels decreased gradually among the petal development. The results showed the expression level of *McCHS* was consistent with the variation of petal color and anthocyanins accumulation in different crabapple cultivars, and the expression intensity of *McCHS* induced the petal pigmentation level in crabapple. In addition, the color variation in ‘Radiant’ petals was induced by the down-regulation of *McCHS* transcriptional level.

**Figure 4:**
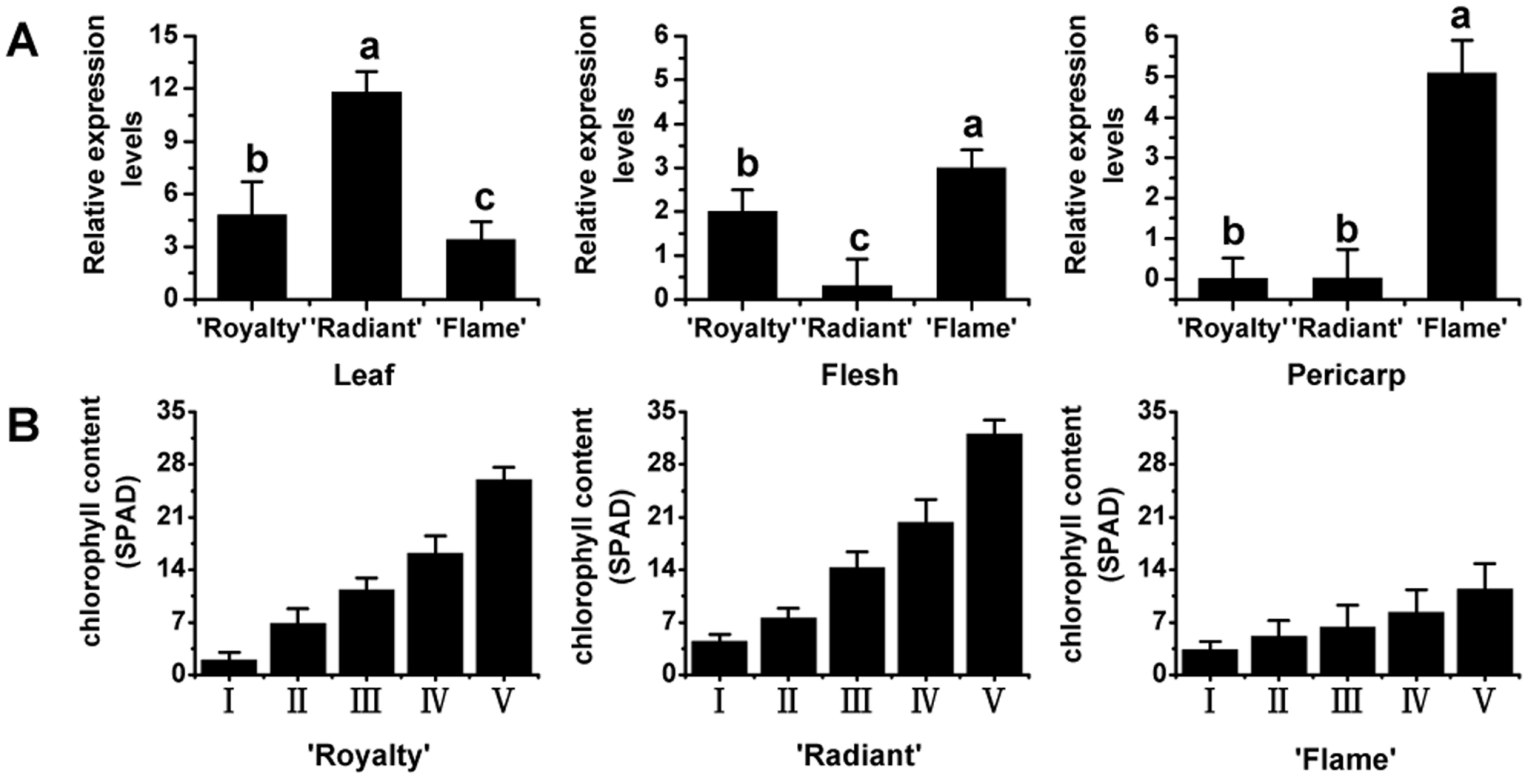
Relative expression profile of *McCHS* in different organs or tissues and chlorophyll content in leaves of the three *Malus* crabapple cultivars: ‘Royalty’, ‘Radiant’ and ‘Flame’. (A) Relative expression profile of *McCHS* in different organs or tissues. (B) The Chlorophyll contents in leaves of the three crabapple cultivars. All real time-PCR reactions were normalized using the Ct value corresponding to a *Malus* crabapple 18S ribosomal RNA gene (DQ341382). Similar material was collected at the same time from the three cultivars. Leaves were all collected at the time of petal stage IV (Figure 2) and the fruits were collected at a fully ripe stage. The Chlorophyll contents of leaves on the tree in vivo were measured by Chlorophyll Content Meter (CL-01, Hansatech, UK). Five Phyllotaxy was measured according to the order of the leaves, respectively. Error bars correspond to the standard error of the mean ± SE of three replicate reactions. Different letters above the bars indicate significantly different values (*P*<0.05) calculated using one-way analysis of variance (ANOVA) followed by a Duncan's multiple range test.

The expressions of *McCHS* in leaves and fruits at maturity were also investigated in the three cultivars. The phenotypes of leaf, fruit flesh and fruit pericarp are shown in Figure S1. When comparing leaves, the highest expression of *McCHS* was in ‘Radiant’, with a value more than twice that of ‘Royalty’ and four times that of ‘Flame’ (Figure 4A). *McCHS* transcripts were most abundant in the flesh and pericarp of ‘Flame’ fruits, but they were almost undetectable in the pericarp of ‘Royalty’ and ‘Radiant’ (Figure 4A). In fruit flesh, the expression profile of *McCHS* showed an opposite trend from that of leaves (Figure 4A). These results suggest that the expression of *McCHS* have different patterns in different tissues/organs among these three cultivars, so we deduced that the expression of *McCHS* may be also involved in other flavonoids compounds biosynthesis. As an important factor to determine the leaf color, the chlorophyll contents in leaves from the three cultivars were measured at five development stages, the results suggested that the chlorophyll content was higher in ever-red-leaf ‘Royalty’ leaves than spring-red-leaf ‘Radiant’ and ever-green-leaf ‘Flame’, and gradually decreased with the development of leaves in crabapple. So the red leaf color of crabapple is depended on the accumulation of anthocyanin and expression of *McCHS*, not the chlorophyll content (Figure 4B).

### RNA expression profiles of flavonoid biosynthetic genes in *Malus* crabapple petals

**Figure 5:**
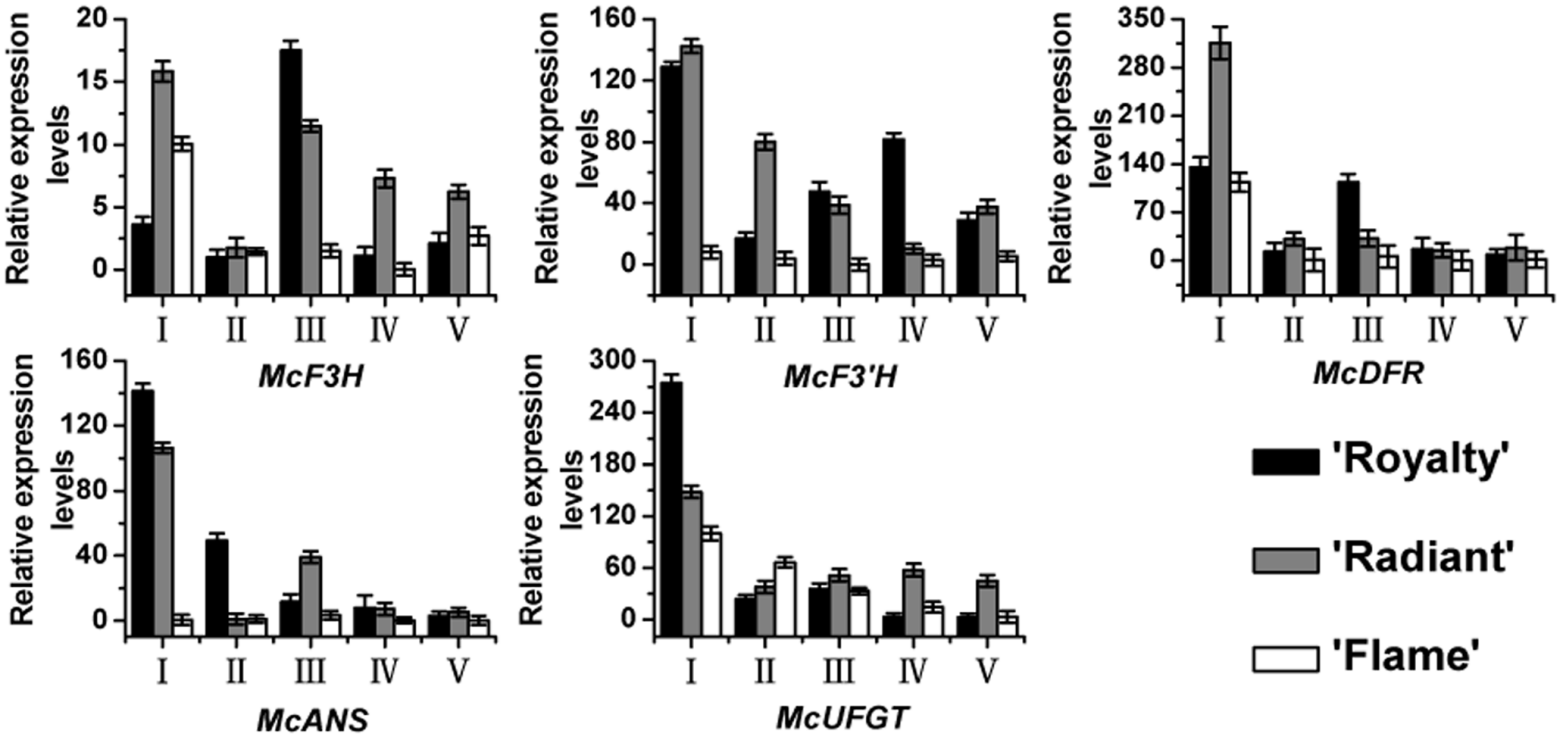
Relative expression profile of downstream genes of *McCHS* during flower developmental stages. Real-time PCR was used to analyze the expression patterns of *McCHS*, *McF3H*, *McF3′H*, *McDFR*, *McANS* and *McUFGT* in petals of *Malus* cultivars ‘Royalty’ and ‘Radiant’, ‘Flame’. All real time-PCR reactions were normalized using the Ct value corresponding to a *Malus* crabapple 18S ribosomal RNA gene (DQ341382). Stages referred to on the x axis are: (I) 6 days before full bloom; (II) 3 days before full bloom; (III) 1 day before full bloom; (IV) full bloom; and (V) 3 days after full bloom. Error bars correspond to the standard error of the mean ± SE of three replicate reactions.

To further confirm how *CHS* regulated the other anthocyanin biosynthetic genes to affect anthocyanin biosynthesis, we analyzed the expression of anthocyanin biosynthetic genes that are located downstream of *McCHS* (Figure 5). The expression levels of the downstream genes *McF3H*, *McF3′H*, *McDFR*, *McANS* and *McUFGT* were almost all higher in ‘Royalty’ and ‘Radiant’ petals than in ‘Flame’ petals at all developmental stages, with an exception being *McUFGT* at stage II. To some extent, the expression patterns of *McF3H*, *McF3′H* and *McDFR* showed a similar pattern among the three *Malus* crabapple cultivars and was closely related to flavonol levels (Figure 2E and 5). While the expression of *McF3H*, *McF3′H* and *McDFR* in ‘Radiant’ and ‘Flame’ petals was similar to *McCHS*, but not in ‘Royalty’. Most importantly, *McCHS* showed the same spatial and temporal expression pattern as *McANS* and *McUFGT* in all three cultivars. Taken together, the results of transcriptional level suggested *McCHS* gene regulate the flavonoid biosynthetic genes to color the plants.

### Quantitative PCR expression analysis of putative transcription factor genes in *Malus* crabapple petals

**Figure 6:**
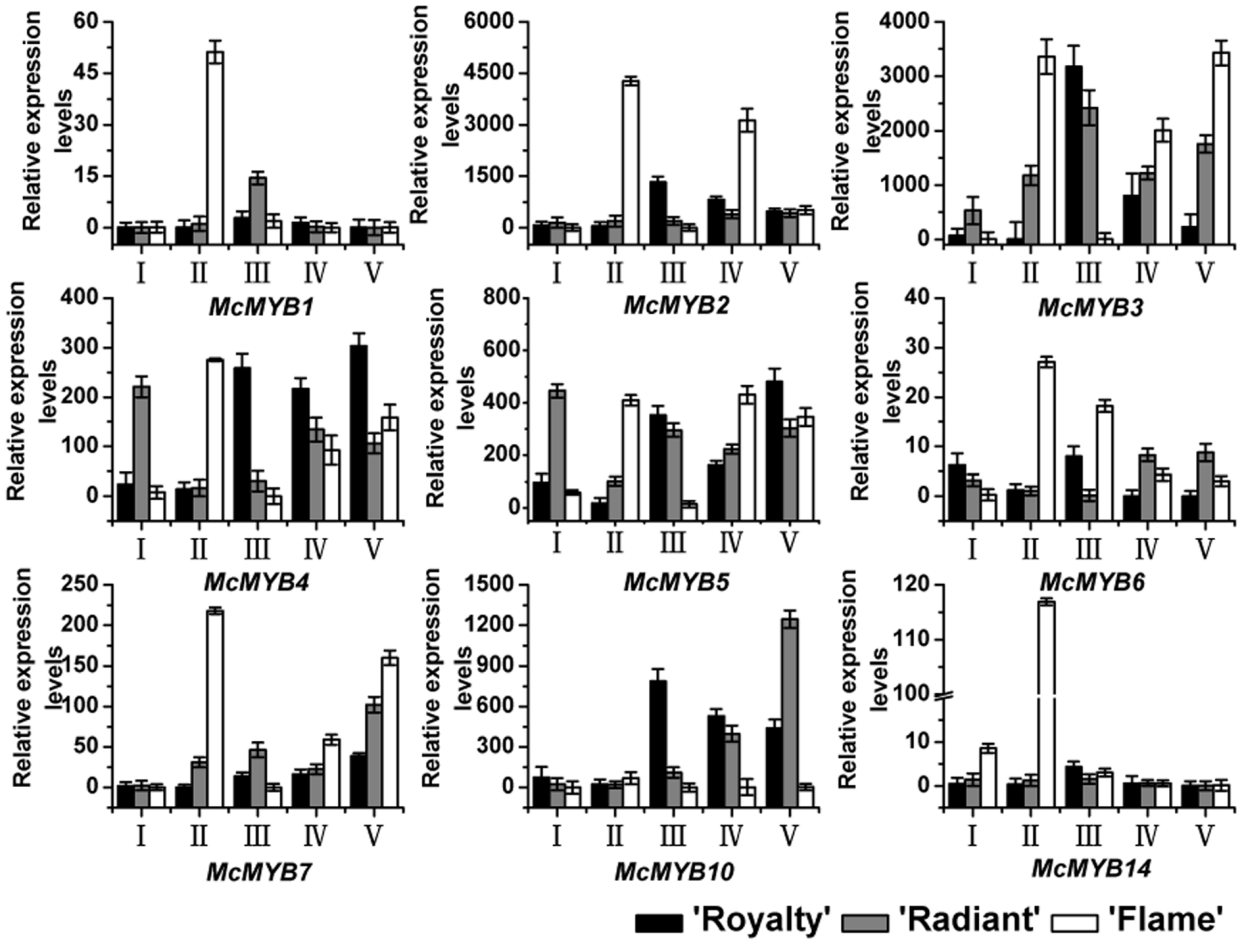
Relative expression profiles of potential MYB flavonoid activators during flower development. Real-time PCR was used to analyze the transcripts in petals of *Malus* cultivars ‘Royalty’ and ‘Radiant’, ‘Flame’, and all real time-PCR reactions were normalized using the Ct value corresponding to a *Malus* crabapple 18S ribosomal RNA gene (DQ341382). Stages referred to on the x axis are: (I) 6 days before full bloom; (II) 3 days before full bloom; (III) 1 day before full bloom; (IV) full bloom; and (V) 3 days after full bloom. Error bars correspond to the standard error of the mean ± SE of three replicate reactions.

Transcriptional control of flavonoid biosynthesis is one of the best characterized regulatory systems in plants, and involves integrating both developmental and various biotic and abiotic stress signals and the promoters of flavonoid biosynthetic genes via the control of TFs [37]–[38]. Previously, we showed that anthocyanin biosynthetic genes are regulated by three classes of TFs [14]–[16]. To explore the transcriptional regulatory mechanisms of *McCHS*, we investigated the expression profiles of nine MYB TFs during the five petal developmental stages (Figure 6). For all three crabapple cultivars, the expressions of *McMYB1*, *McMYB2* and *McMYB14* were barely detected in petals, except at stage II. The expression levels of *McMYB3* and *McMYB7* showed a very similar profile in petals from the same cultivar and almost increaed with the development of crabapple petals. Furthermore, *McMYB4* expression displayed a similar pattern to that of *McMYB5* in petals from the same cultivars and decreased at the early development stages and increased at later development stages of crabapple petals. The profiles of *McMYB6* and *McMYB10* transcript accumulation suggested more specialized expression patterns between ‘Royalty’, ‘Radiant’ and ‘Flame’ petals. In ‘Royalty’ and ‘Flame’, the expressions of *McMYB6* and *McMYB10* were increased at the early development stages and decreased at later development stages of crabapple petals, but the transcriptions of *McMYB6* and *McMYB10* were increased with the development of petals in ‘Radiant’.

*McCHS* showed decreased expression in ‘Royalty’ petals during development, while the expression of *McMYB4* increased over the same period, revealing a negative correlation between the *McMYB4* and genes in the flavonoid biosynthetic pathway, and the anthocyanins accumulation was negatively regulated by *McMYB4*. In ‘Radiant’ petals, the expression of *McMYB5* was almost consistent with the expression pattern of *McCHS* and the contents of anthocyanins, flavonol (kaempferol, quercetin and rutin) and apigenin. In ‘Flame’ petals, the expression of *McCHS* also showed a positive correlation with *McMYB5* expression (except for the second petal development stage). Meanwhile, the expression trend of TFs and *McCHS* were consistent with the accumulation of kaempferol, quercetin, rutin, apigenin and catechin, which were the main flavonoids compounds in white petal cultivar ‘Flame’. These data are consistent with *McMYB4, McMYB5* that may be involved in the regulation of *McCHS* expression during petal development in these cultivars, and these results suggested that due to different accmulation of flavonoids compounds, the *McCHS* gene have different biosynthesis functions and regulated by different transcription factors.

### Overexpression *McCHS* in tobacco plants

**Figure 7:**
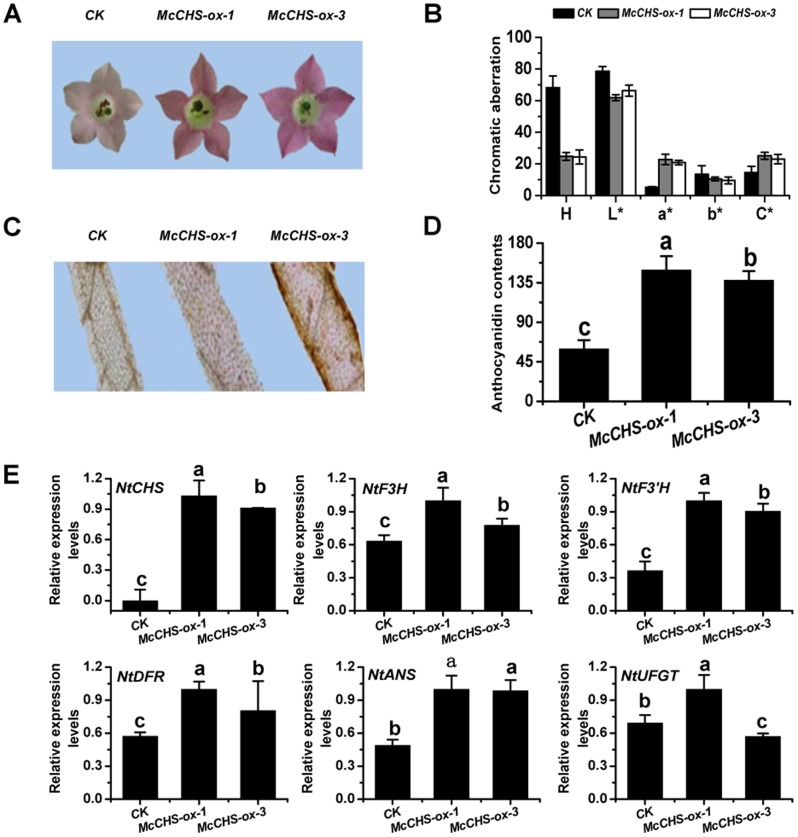
Phenotypic analysis of *McCHS* overexpressing transgenic tobacco flowers and expression profiles of target genes. (A) Typical flower phenotypes of control lines (*CK*) and *McCHS-ox-1 and McCHS-ox-3* tobacco plants overexpressing *McCHS*. (B) Petal color of control plants (*CK*) and *McCHS-ox-1* and *McCHS-ox-3*. (C) Microscopic observation of the transgenic tobacco petals. (D) Content of total anthocyanin in petals of control lines (*CK*) and *McCHS-ox-1* and *McCHS-ox-3*. (E) Relative expression profiles of endogenous anthocyanin biosynthesis genes in transgenic tobacco flowers. A spectrophotometric colorimeter was used to measure color changes in petals and HPLC was used to analyze the total anthocyanin content in the petals of transgenic tobacco. Real-time PCR was used to assess the expression of target genes (*NtCHS*, *NtF3H*, *NtF3′H*, *NtDFR*, *NtANS*, *NtUFGT*) in *McCHS-ox* tobacco plants. *CK* refers to wild type tobacco. All real time-PCR reactions were normalized using the Ct value corresponding to the *NtActin* gene (GQ339768). Error bars correspond to the standard error of the mean ± SE of three replicate reactions. Analysis of relative expression levels of control and *McCHS-ox* lines and different letters above the bars indicate significantly different values (*P*<0.05) calculated using one-way analysis of variance (ANOVA) followed by a Duncan′s multiple range test.

To investigate the temporal and spatial expression of *McCHS* in plants, transgenic tobacco plants expressing *McCHS* under the control of a constitutive CaMV35S promoter were generated, and two independent T2 lines were characterized. Compared to wild type, tobacco plants transformed with *35S::McCHS* developed a more intense pigmentation in the petals, especially in *McCHS-ox-1* line (Figure 7A). The lightness value L*, hue value a*, hue value b* of the *McCHS*-overexpressing tobacco petals were also measured. The ‘H’ value (hue angle) decreased to one-third of that of control plant petals and the ‘a*’ values were 4.0 and 5.0-fold higher than in control plant petals, indicative of a stronger red color (Figure 7B). In addition, we confirmed by microscopy that more anthocyanins accumulated in the petal cells of the transgenic tobacco lines (Figure 7C).

To confirm the biochemical activity of *McCHS*, anthocyanins content and the expression level of the *McCHS* gene were detected in the transgenic tobacco petals, (Figure 7D). The anthocyanins content of petals was approximately 3-fold greater in the *McCHS-ox-1* line and 2.5-fold greater in the *McCHS-ox-3* line compared to control petals. These results are consistent with the visual flower color phenotypes.

**Figure 8:**
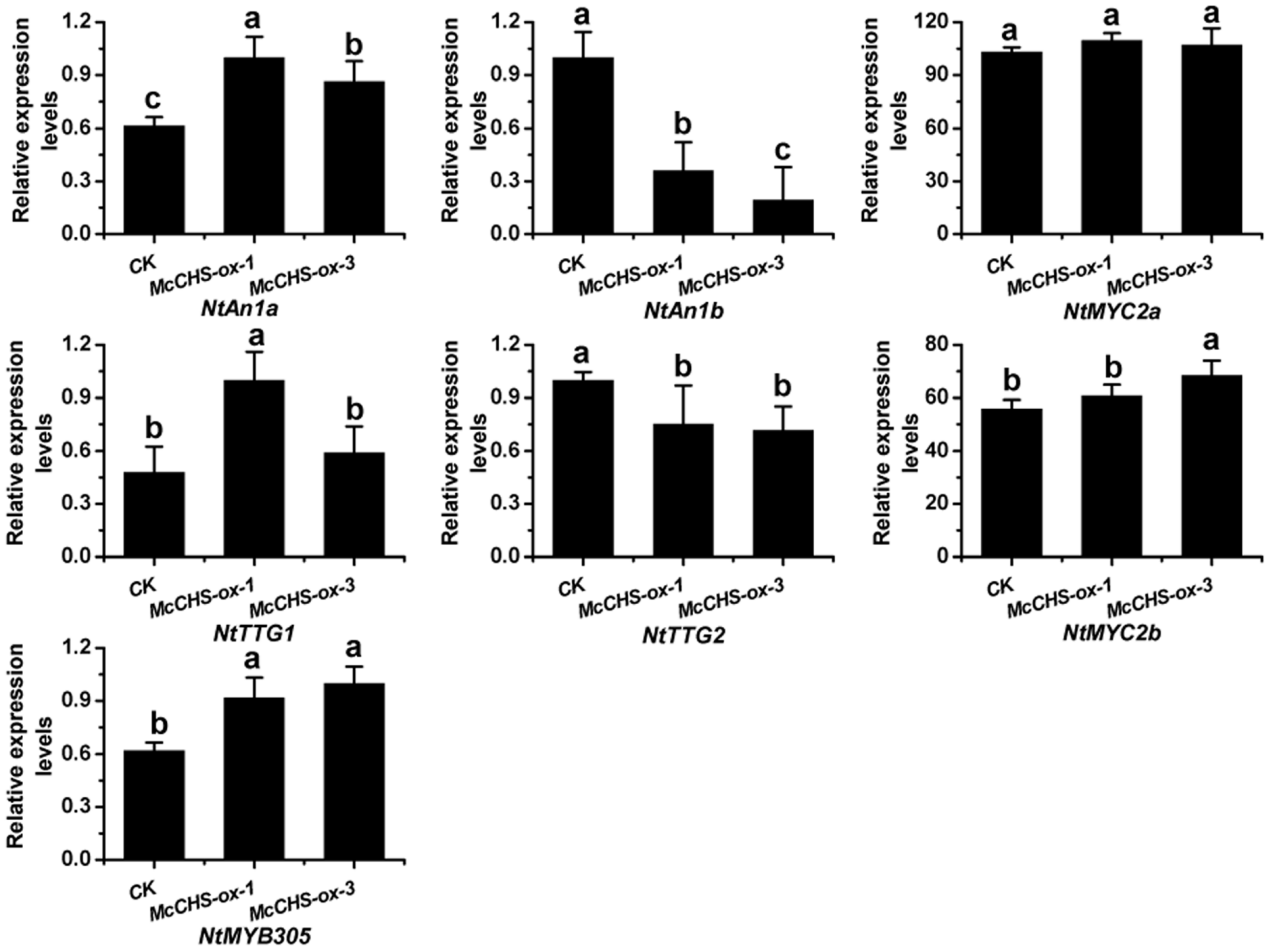
Relative expression profiles of endogenous tobacco anthocyanin regulation factors during flower development. Real-time PCR was used to analyze the transcripts in transgenic tobacco flowers, and all real time-PCR reactions were normalized using the Ct value corresponding to the *NtActin* gene (GQ339768). Error bars correspond to the standard error of the mean ± SE of three replicate reactions. Analysis of relative expression levels of control and *McCHS-ox* lines and different letters above the bars indicate significantly different values (*P*<0.05) calculated using one-way analysis of variance (ANOVA) followed by a Duncan's multiple range test.

The qRT-PCR results confirmed a massive elevation of *McCHS* transcript levels in the transgenic lines, and the absence of expression in the control plants, as expected. The accumulation of anthocyanins was consistent with the relative expression profile of *McCHS* in *McCHS*-overexpressing tobacco petals. Moreover, the over-expression of *McCHS* in tobacco significantly promoted the expression of the downstream endogenous genes *NtF3H, NtF3′H*, *NtDFR*, *NtANS*, and *NtUFGT* (Figure 7E). To further explore the molecular regulation mechnism in transgenic tobacco, we tested the expression of several endogenous tobacco anthocyanin regulation factors. Interestingly, the results showed the massive accumulated *McCHS* can alter the expressions of these transcription factors. The expression levels of *NtAn1a*, *NtTTG1*, *MYB305*, *NtMYC2a* and *NtMYC2b* were increased, and *NtAn1b* and *NtTTG2* were decreased by overexpressed *McCHS* (Figure 8).

## Discussion

*CHS*, which catalyzes the first committed step in anthocyanin biosynthesis, plays a central role and provides a common chalcone precursor for the production of all intermediates and final products of the flavonoid biosynthetic pathway. Many studies have analyzed the activity of *CHS* in different plants, including *P. hybrida* [17], *Antirrhinum majus* [39], maize [40], *Arabidopsis* [41], strawberry and tomato [32], [42] and apple [33]. The first characterized *chs* mutant, the light white mutant of *A. majus*, whose single *CHS* gene was knocked out, was reported to lack anthocyanins and UV-absorbing flavonoids [39]. This mutant established a relationship between CHS activity and the white-flowered phenotype. In maize, recessive mutations in both *CHS* genes of maize, *C2 and Whp*, results in a white pollen phenotype and male sterility [26], [40]. Moreover, down-regulation of *MdCHS* was reported to lead to major changes in plant development, resulting in plants with shortened internode lengths and leaf areas, and a greatly reduced growth rate, as well as a loss of anthocyanins, tannins and phenylpropanoid-related coloration of the stem and fruit skin [33]. But the important role of *CHS* in petal coloration level in different cultivars is still unknown.

In this current study, we evaluated the expression of *McCHS*, associated downstream genes and several MYB TFs in three different *Malus* crabapples cultivars, and determined that *McCHS* shows different expression patterns in various tissues and organs. A positive correlation is identified between the expression of *McCHS* and the accumulation of anthocyanins in petals, but not in other organs, indicated that the the petal coloration level is determined by the expression level of *McCHS* specially, the more expression of *McCHS* has more red color in crabapple petals. Meanwhile, the expression of *McCHS* is responsible for the color fade process during petal expansion in color variation cultivar. The different expression patterns of this gene in accumulation during leaves, petals and fruits development in different cultivars, and different tissues/organs, which may be explained by diversity flavonoids compounds accumulation during leaves, petals and fruits development in different cultivars, and is regulated by various organ-specific transcription factors. In nectarine, *CHS* expression was reported that the expression in the skin was much higher than that in the fruit flesh [36]. The same result was found in apple, the expression of *CHS* was much higher in the fruit skin than in flesh and green leaves [43]. To sum up, *CHS* expression was higher in red tissues/organs than in colorless tissues/organs. Several reports showed that *ANS* [44]–[46] and *UFGT* [44], [47]–[49] were two key anthocyanin biosynthetic pathway genes. A positive correlation between the expression of *McCHS* and its downstream genes *McANS*, *McUFGT* was observed. So this is additional evidence that *McCHS* gene is involved in anthocyanin biosynthesis. Furthermore, the expression patterns of the downstream genes *McF3H*, *McF3′H* and *McDFR* shows similar pattern to *McCHS* in ‘Radiant’ and ‘Flame’ petals. This may suggest that the transcript abundance of *McCHS* may influence the expression of other anthocyanin biosynthetic genes, and thus regulate the production of anthocyanin compounds, or they may be regulated by the same TFs.

*NtAn1a*, *NtAn1b*, *NtTTG1*, *NtTTG2*, *MYB305*, *NtMYC2a* and *NtMYC2b* have been proved to play an important role in tobacco anthocyanin biosynthesis pathway [50]–[55]. In our results, overexpressed *McCHS* can alter the expression of endogenous tobacco anthocyanin regulation factors. So we presume that massive expression of *McCHS* may be as a feedback signal to activate or inhibit the anthocyanin-related transcription factors, resulting in enhanced anthocyanins accumulation in tobacco.

The expression of *CHS* is not only responsible for the anthocyanins biosynthesis, but also for the flavonol and other flavonoids componunds biosynthesis in plants. So the transcription level of *McCHS* is different in various crabapple cultivars, and may be regulated by several MYB transcription factors. We identify a close relationship between *McCHS* and *McMYB4*, *McMYB5*. The three cultivars show individual patterns of correlated expression and some of the results support the conclusions resulting from previously published data. MYB4 functions as a transcription repressor which involved in the regulation of diversity secondary metabolism, such as anthocyanins, lignin, flavonol, proanthocyanidin, and as a target gene that the transcript level of *CHS* was regulated by MYB4 in *A. thaliana*, turnip, Kiwifruit, *Pinus taeda* and so on [56]–[60]. The transcript levels of *MYB5* and all proanthocyanidin-specific genes were previously shown to be down-regulated, while anthocyanin-specific gene expression increased, leading to a switch from proanthocyanidin biosynthesis to anthocyanin biosynthesis in the mature phase of grape berries [61]–[64]. Transient expression assays have shown that VvMYB5a and VvMYB5b could activate several grapevine flavonoid pathway genes and affect the biosynthesis of anthocyanins, flavonols, tannins and lignins in reproductive organs when overexpressed in tobacco (*Nicotiana tabacum*), including *CHS* gene [65]–[66]. Meanwhile, the promoter of *McCHS* has several MYB-binding cis-elements and maybe regulated by MYB TFs [67]. All of these informations support our observation in crabapple that *McCHS* can be regulated by these transcription factors in different crabapple cultivars due to the various flavonoids compounds accumulation (Figure 2E, Figure 3C and Figure 6).

To confirm that the accumulation of flavonoids was influenced by expression of the *McCHS* gene, we over-expressed *McCHS* under the control of a constitutive CaMV35S promoter in tobacco and observed an increase in anthocyanins accumulation, as well as increased expression of downstream endogenous tobacco anthocyanin biosynthesis genes. This may indicate that massive expression of *McCHS* may regulate the downstream genes to promote anthocyanin accumulation. It has also been shown that overexpression of *CHS* can lead to an accumulation of flavonoids. Such as, overexpression of a *CHS* gene in *Linum usitatissimum*, tomato and potato (*Solanum tuberosum*) resulted in an increase in total phenolic compounds in *Linum usitatissimum* [68], an accumulation of naringenin in the tomato fruit flesh and an accumulation of anthocyanins in potato tubers, respectively [69]. Besides overexpression, silencing of *CHS* gene expression in several plants has been shown to result in reduced anthocyanin levels in flowers. *CHS* gene silencing in tobacco [70], chrysanthemum [71], *P. hybrida* [17], [72], carnation [73], Gutterson rose [73], lisianthus [74] and gentian [75] resulted in plants with very pale pink, or entirely white flowers due to reduced anthocyanin levels, which again is congruent with the results reported here for *Malus* crabapple.

The expression of *CHS* is regulated by several factors, such as environmental conditions, the variation in the conditions in plants (such as pH), transcription factors and so on [12], [76]–[79]. Recently, the report showed that the different methylation levels of *OgCHS* promoter altered the expression of this gene in different *Oncidium* orchid cultivars [19]. Therefore, epigenetic modification is a new sight that it can affect the anthocyanin biosynthetic gene expression. Future study is required on the possible regulation mechanism controlling *McCHS* expression in crabapple.

## Conclusions

In this study, we have shown evidences that the expression level of *McCHS* is consistent with the variation of petal color and anthocyanins accumulation in different crabapple cultivars, and the expression intensity of *McCHS* determined the petal pigmentation level in crabapple specially. On the other hand, the expression of *McCHS* is responsible for the color fade process during petal expansion in color variation cultivar. The regulation mechanism of *McCHS* expression is complicated, and several transcription factors might be involved in regulating *McCHS* expression in crabapple petals. Key future questions that need to be addressed include but not limited to the transcription regulation and epigenetic modification of *McCHS* expression. Undoubtedly the work described in this report will trigger a series of exciting future research projects to elucidate the mechanisms governing plant coloration.

## Materials and Methods

### Plant materials

The petals of *Malus* crabapple *cv*. ‘Royalty’, ‘Radiant’ and ‘Flame’ were collected at different development stages (I, six days before full bloom; II, three days before full bloom; III, one day before full bloom; IV, full bloom V, three days after full bloom). These trees were grown in the Crabapple germplasm of Beijing University of Agriculture (Changping District, Beijing, China) received standard horticultural practices, and disease and insect control. Control and T2 transgenic tobacco (*Nicotiana benthamiana*) plants were grown in a greenhouse. Flowers were collected for studies at the full-bloom stage (IV). The leaves of *Malus* crabapple *cv*. ‘Royalty’, ‘Radiant’ and ‘Flame’ were collected at the same time with petals at the full bloom stage. Fruits were collected at their fully mature stage in October 2012. At sampling time all the plant materials were immediately frozen in liquid nitrogen and stored at -80°C until RNA or phenolic compounds were extracted.

### Color analysis

For flower color evaluation, color components of the CIE L*a*b* coordinate, namely lightness and hue, were measured immediately after flowers were picked with a hand spectrophotometer (Konica Minolta CR-400, Minolta, Japan, Tokyo, Japan). The ‘L*’ value represents brightness and darkness, the ‘a*’ value represents greeness and redness as the value increases from negative to positive, and ‘b*’ represents blueness and yellowness. The ‘C*’ value represents chroma, calculated according to C* = 1/2(a*2+b*2). The hue angle (H) was calculated according to the following equation: H = arctan (b*/a*) [80]. Three areas of the petal adaxial surface were subjected to color measurement. Values were obtained and averaged from three replicate petals at 5 stages from three different flowers. For leaf color evaluation, the Chlorophyll contents of leaves on the tree in vivo were measured by Chlorophyll Content Meter (CL-01, Hansatech, UK). The same part of leaf adaxial surface was subjected to color measurement. Five Phyllotaxy was measured according to the order of the leaves, respectively. Values were obtained and averaged from three replicate leaves of five Phyllotaxy from three different cultivars.

### Identification and quantification of anthocyanins and flavonols

Pigments were quantified by high-performance liquid chromatography (HPLC). One hundred milligrams of petals were homogenized with a mortar and pestle in liquid nitrogen, then 1 ml of extraction solution (methanol: water: formic acid: trifluoroacetic acid [70∶27∶2∶1, v/v]) was added and the mixture was stored overnight at 4°C in the dark. The mixture was then centrifuged at 4°C at 12,000×g for 15 min, and the supernatants were filtered through a 0.22 µm Millipore filter (Billerica, MA, USA). Anthocyanins were identified and quantified using an HPLC1100-DAD system (Agilent Technologies, Waldbronn, Germany). Detection was performed at 520 nm for anthocyanins, 350 nm for flavonoids. A NUCLEODURH C18 column (250 mm64.66 mm) (Pretech Instruments, Sollentuna, Sweden) operating at 25°C was used for separation and compounds were eluted in a mobile phase consisting of solvent A, (trifluoroacetic acid: formic acid: water [0.1∶2∶97.9]) and solvent B (trifluoroacetic acid: formic acid: acetonitrile: water [0.1∶2∶35∶62.9]) at a flow rate of 0.8 ml min^−1^. The elution program followed the procedure described by Wu and Prior [81] with some modifications. Solvent B was initially 30% and increased linearly in steps to 35% at 5 min, 40% at 10 min, 50% at 30 min, 55% at 50 min, 60% at 70 min, 30% at 80 min. HPLC analysis was performed as described in Ohno [82].

### RNA extraction and Quantitative real-time PCR analysis

Total RNA was extracted from flowers, leaves and fruit flesh and pericarp using an RNA Extract Kit (Aidlab, Beijing, China), according to the manufacturer's instructions. DNase I (TaKara, Japan) was added to remove genomic DNA and the samples were subjected to cDNA synthesis using the Access RT-PCR System (Promega, USA) according to the manufacturer's instructions. The expression levels of *McCHS*, *McF3H*, *McF3′H*, *McDFR*, *McANS*, *McUFGT*, *McMYB1*, *McMYB2*, *McMYB3*, *McMYB4*, *McMYB5*, *McMYB6*, *McMYB7*, *McMYB10*, *McMYB14* were analyzed using quantitative real-time PCR (qRT-PCR) with SYBR Green qPCR Mix (Takara, Japan) and a Bio-Rad CFX96 Real-Time PCR System (BIO-RAD, USA), according to the manufacturers’ instructions. The expression levels of *NtCHS*, *NtF3H*, *NtF3′H*, *NtDFR*, *NtANS* and *NtUFGT* were analyzed using the same techniques. Primers are designed by NCBI Primer BLAST and listed in Table S1. *Malus* ribosomal 18S rRNA gene (DQ341382) was used as a reference gene for the target genes *McCHS*, *McF3H*, *McF3′H*, *McDFR*, *McANS*, and *McUFGT, McMYB1*, *McMYB2*, *McMYB3*, *McMYB4*, *McMYB5*, *McMYB6*, *McMYB7*, *McMYB10*, *McMYB14*; *NtActin* (GQ339768) was used as a reference gene for the target genes *NtCHS*, *NtF3H*, *NtF3′H*, *NtDFR*, *NtANS*, *NtUFGT*. The resulting cDNA samples for reverse transcription were serially diluted (1, 1/10, 1/100, 1/1000, 1/10000). Real-time RT-PCR analysis was carried out in a total volume of 20 µl, containing 9 µl of 2×SYBR Green qPCR Mix (Takara, Japan), 0.1 µM of each specific primer, and 100 ng of template cDNA. The reaction mixtures were heated to 95°C for 30 s, followed by 39 cycles at 95°C for 10 s, 59°C for 15 s, and 72°C for 30 s. A melting curve was generated for each sample at the end of each run to ensure the purity of the amplified products. Standard dilution curves were performed for each gene fragment, and all data were normalized to the level of the reference gene transcript. Primers for real-time experiments were designed using primer premier v5.0 software with forward and reverse primers corresponding to two different exons.

### Phylogeny and sequence alignment

Protein consensus sequences were determined from the coding sequences of *CHS* and aligned with translated genome reference sequences and published CHS protein sequences from other species with DNAMAN 5.2.2. A phylogenetic tree was produced using MEGA version 5.10 [83] based on the coding sequence alignment of chalcone synthase-like genes, using a minimum evolution phylogeny test and 1,000 bootstrap replicates.

### Expression vector construction and tobacco transformation

The entire *McCHS* coding sequence was amplified by PCR with the *McCHS-F* and *McCHS-R* primers, using cDNA from ‘Royalty’ petals as a template. The primers for *McCHS* amplification contained *Spe*I and *Kpn*I restriction enzyme sites, which were used to clone the coding region of the *McCHS* gene into the pBI121 vector. This was then used to transform *Agrobacterium* strain LBA4404 which in turn was used to transform tobacco (*N. benthamiana*) Wisconsin 38 using the leaf disk method [84]. Transgenic plants were selected based on kanamycin resistance. T2 progeny from the transgenic plants was used for further analysis and compared to wild-type non-transformed lines grown under the same conditions. All the primers used are listed in Table S1.

### Statistical analysis

Statistical analysis and graphing was carried out using the OriginPro 8 statistical software (OriginLab Corporation, USA). Microsoft Office PowerPoint 2003 was used for artwork. Error bars represents mean ± SE of three replicate reactions.

## Supporting Information

Figure S1 **The phenotypes of different organs of the three** ***Malus*** **crabapple.**
(DOC)

Table S1 **Sequences of primers and relevant accession numbers.**
(DOC)
