# Supplementary material for: A Malus Crabapple Chalcone Synthase Gene, McCHS, Regulates Red Petal Color and Flavonoid Biosynthesis
Source: PLoS One. 2014 Oct 30;9(10):e110570. doi: 10.1371/journal.pone.0110570 (PMC4214706; doi:10.1371/journal.pone.0110570)
Supplement: Figure S1 — The phenotypes of different organs of the three Malus crabapple. (DOC) [file pone.0110570.s001.doc]

**Supporting Information**

**Figure S1. The phenotypes of different organs of the three *Malus* crabapple.**


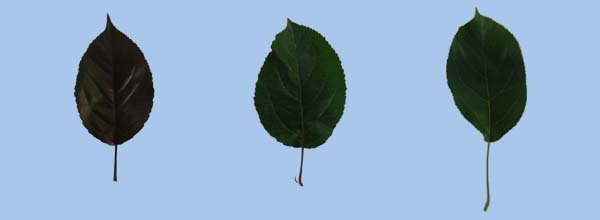


**‘Royalty’ ‘Radiant’ ‘Flame’**


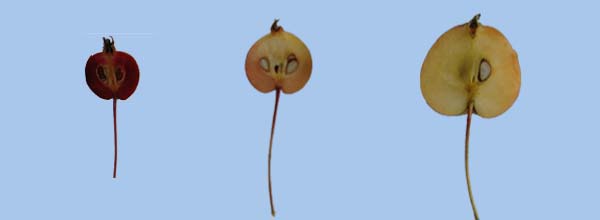


**‘Royalty’ ‘Radiant’ ‘Flame’**


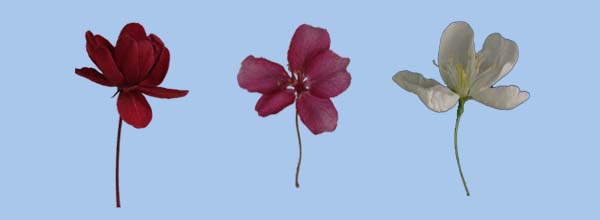


**‘Royalty’ ‘Radiant’ ‘Flame’**


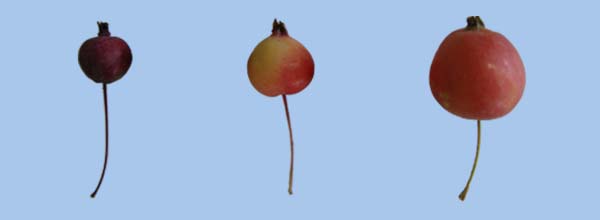


**‘Royalty’ ‘Radiant’ ‘Flame’**
