# Supplementary material for: A Malus Crabapple Chalcone Synthase Gene, McCHS, Regulates Red Petal Color and Flavonoid Biosynthesis
Source: PLoS One. 2014 Oct 30;9(10):e110570. doi: 10.1371/journal.pone.0110570 (PMC4214706; doi:10.1371/journal.pone.0110570)
Supplement: Table S1 — Sequences of primers and relevant accession numbers. (DOC) [file pone.0110570.s002.doc]

**Supporting Information**

**Table S1. Sequences of primers and relevant accession numbers.**

| Accession number | ID | Primer Sequences (5’-3’) | Purpose |
| --- | --- | --- | --- |
| FJ599763 | McCHS-F | TGACCGTCGAAGTTCGC | qRT-PCR |
|  | McCHS-R | TTTGTCACACATGCGCTGGA | qRT-PCR |
| FJ817486 | McF3H-F | ACGAAGACGAGCGTCCAAAG | qRT-PCR |
|  | McF3H-R | CTCCTCCGATGGCAAAGCAA | qRT-PCR |
| KF481684 | McF3'H-F | CGTTGCTGTCGCTCACGGATGA | qRT-PCR |
|  | McF3'H-R | ATGACGTGTCAGTGCCAGCTGTG | qRT-PCR |
| FJ817487 | McDFR-F | CCGAGTCCGAATCCGTTTGT | qRT-PCR |
|  | McDFR-R | CCTTCTTCTGATTCGTGGGGT | qRT-PCR |
| FJ817488 | McANS-F | CACAGGGGCATGGTGAACAA | qRT-PCR |
|  | McANS-R | TTCACTTGGGGAGCAAAGCC | qRT-PCR |
| KF495603 | McUFGT-F | CGGGTGCAAATTCGGACCAAGGGT | qRT-PCR |
|  | McUFGT-R | ACGACCCATCTCGCTCGTCCCTCG | qRT-PCR |
|  | McMYB1-F | GCTCACACCAACAAAGGAGC | qRT-PCR |
|  | McMYB1-R | GCAGCTCTTCCCACATCGAA | qRT-PCR |
|  | McMYB2-F | GCTGCGATAAGCAGGACACAA | qRT-PCR |
|  | McMYB2-R | ACCGGTTCCCTAGGAGTGC | qRT-PCR |
|  | McMYB3-F | ACATTAAGACTCATGGGGAAGGC | qRT-PCR |
|  | McMYB3-R | GCAGCCTTCCAGCAATCAAC | qRT-PCR |
| JX013493 | McMYB4-F | GGACCAGCAGCAGGAAACTA | qRT-PCR |
|  | McMYB4-R | ACAACCCTCCATTAATGCCGAC | qRT-PCR |
|  | McMYB5-F | GACGCCGTGTTGCGTAAAG | qRT-PCR |
|  | McMYB5-R | GACGCCGTGTTGCGTAAAG | qRT-PCR |
|  | McMYB6-F | AGACTGCCGGGACGAACCGA | qRT-PCR |
|  | McMYB6-R | CCTTGTCGCTTCCTTGCTCCGT | qRT-PCR |
|  | McMYB7-F | AGGGTGCAAAAACAGGCGCG | qRT-PCR |
|  | McMYB7-R | CGGCACCCAGAAACCCCGAAC | qRT-PCR |
| JX162681 | McMYB10-F | GGCGCATGATCTTGGCGACAGT | qRT-PCR |
|  | McMYB10-R | ACGCCACCACAAACGTCGTCG | qRT-PCR |
|  | McMYB14-F | GCTGGCGTTCTCTCCCCAAGC | qRT-PCR |
|  | McMYB14-R | TCGTCCTTGCTGAAGGGGCCT | qRT-PCR |
| DQ341382 | 18SR-F | GTCACTACCTCCCCGTGTCA | qRT-PCR |
|  | 18SR-R | GAGCCTGAGAAACGGCTACC | qRT-PCR |
| FJ599763 | McCHS-F | GCGCTACTAGTATGGTGACCGTCGAAGAAGTTC | pBI121-McCHS |
|  | McCHS-R | GTATGG TACCTCAAGCACCCACACTGTG | pBI122-McCHS |
| AF311783 | NtCHS-F | CATGGCACCTTCCCTTGATGC | qRT-PCR |
|  | NtCHS-R | GGGCTGGCCCCATTCTTTGA | qRT-PCR |
| AF036169 | NtF3H-F | GGCACCTTCGACATTGACAG | qRT-PCR |
|  | NtF3H-R | CCACTTTTGGACGCTCATCTTC | qRT-PCR |
| AB289449 | NtF3'H-F | GGACACGTCCTCAAGCACAG | qRT-PCR |
|  | NtF3'H-R | CACGAGCCGGTTCTTTCCAAC | qRT-PCR |
| AB289448 | NtDFR-F | GTTCACGCTACTGTTCGTGATCC | qRT-PCR |
|  | NtDFR-R | CAAGTCCGCTTTCCACAGCG | qRT-PCR |
| AB723683 | NtANS-F | GTGCGACTTATCCATCTGGCC | qRT-PCR |
|  | NtANS-R | CCAGCCCAATAGAAAGCACTGC | qRT-PCR |
| AB723686 | NtUFGT-R | GGTGTTGGATTGTGAAGTGTTG | qRT-PCR |
|  | NtUFGT-F | CCCTTTAGGAAGCCATGCAAC | qRT-PCR |
| HQ589208 | NtAn1a-F | ACCATTCTCGAACACCGAAG | qRT-PCR |
|  | NtAn1a-R | TGCTAGGGCACAATGTGAAG | qRT-PCR |
| HQ589209 | NtAn1b-F | CTTGAACACTTCTCAAACCGA | qRT-PCR |
|  | NtAn1b-R | TGCTAGGGCACAATGTGAAG | qRT-PCR |
| GQ859160 | NtMYC2a-F | TCACCCATTTCTCTCTCTCTCTC | qRT-PCR |
|  | NtMYC2a-R | GAGGTAACAGCAGCAGTAGTAG | qRT-PCR |
| GQ859161 | NtMYC2b-F | TCACCTATTTCTCTCCTCCTCTC | qRT-PCR |
|  | NtMYC2b-R | GTAACAGCAGCAACAGCAGTAG | qRT-PCR |
| FJ346578 | NtTTG1-F | ATGGATAATTCAGCTCCAGATTC | qRT-PCR |
|  | NtTTG1-R | TCAAACTCTAAGGAGCTGCAT | qRT-PCR |
| FJ795022 | NtTTG2-F | AGCGGAGTTGGAAAGGCATCAGG | qRT-PCR |
|  | NtTTG2-R | ATCAATCCCATTAGGCCCAGCAA | qRT-PCR |
|  | NtMYB305-F | CATGGTGAAGGTGTTTGGAA | qRT-PCR |
|  | NtMYB305-F | CATCAGGCCGGAGATAATTT | qRT-PCR |
| GQ339768 | NtActin | CAACGAGCTTCGTGTTGCC | qRT-PCR |
|  | NtActin | CTCTCTGTTGGCCTTTGGGTT | qRT-PCR |
